# CCR2 Contributes to F4/80+ Cells Migration Along Intramembranous Bone Healing in Maxilla, but Its Deficiency Does Not Critically Affect the Healing Outcome

**DOI:** 10.3389/fimmu.2018.01804

**Published:** 2018-08-10

**Authors:** Claudia Cristina Biguetti, Andreia Espindola Vieira, Franco Cavalla, Angélica Cristina Fonseca, Priscila Maria Colavite, Renato Menezes Silva, Ana Paula Favaro Trombone, Gustavo Pompermaier Garlet

**Affiliations:** ^1^Department of Biological Sciences, Bauru School of Dentistry, University of São Paulo, Bauru, Brazil; ^2^Institute of Biological Sciences and Health, Federal University of Alagoas, Maceió, Brazil; ^3^Department of Conservative Dentistry, School of Dentistry, University of Chile, Santiago, Chile; ^4^Department of Endodontics, University of Texas School of Dentistry at Houston, Houston, TX, United States; ^5^Department of Biological and Allied Health Sciences, Universidade do Sagrado Coração, Bauru, Brazil

**Keywords:** osteoimmunology, bone histomorphometry, macrophages, bone healing, intramembranous bone

## Abstract

Bone healing depends of a transient inflammatory response, involving selective migration of leukocytes under the control of chemokine system. CCR2 has been regarded as an essential receptor for macrophage recruitment to inflammation and healing sites, but its role in the intramembranous bone healing on craniofacial region remains unknown. Therefore, we investigated the role of CCR2 on F4/80+ cells migration and its consequences to the intramembranous healing outcome. C57BL/6 wild-type (WT) and CCR2KO mice were subjected to upper right incisor extraction, followed by micro-computed tomography, histological, immunological, and molecular analysis along experimental periods. CCR2 was associated with F4/80+ cells influx to the intramembranous bone healing in WT mice, and CCR2+ cells presented a kinetics similar to F4/80+ and CCR5+ cells. By contrast, F4/80+ and CCR5+ cells were significantly reduced in CCR2KO mice. The absence of CCR2 did not cause major microscopic changes in healing parameters, while molecular analysis demonstrated differential genes expression of several molecules between CCR2KO and WT mice. The mRNA expression of TGFB1, RUNX2, and mesenchymal stem cells markers (CXCL12, CD106, OCT4, NANOG, and CD146) was decreased in CCR2KO mice, while IL6, CXCR1, RANKL, and ECM markers (MMP1, 2, 9, and Col1a2) were significantly increased in different periods. Finally, immunofluorescence and FACS revealed that F4/80+ cells are positive for both CCR2 and CCR5, suggesting that CCR5 may account for the remaining migration of the F4/80+ cells in CCR2KO mice. In summary, these results indicate that CCR2+ cells play a primary role in F4/80+ cells migration along healing in intramembranous bones, but its deficiency does not critically impact healing outcome.

## Introduction

The recruitment of circulating blood monocytes and its transition into macrophages at injured tissues are essential steps of inflammatory immune response and healing processes (1–3). Indeed, macrophages comprise a heterogeneous myeloid cell lineage that participate directly or indirectly in tissue healing by playing a number of functions, such as removing debris and dead cells after injury, as well as producing a large range of growth factors, immunological molecules, and proteolytic enzymes (3–5). However, while these macrophages beneficial contributions to tissue healing are well defined in soft tissue healing (1), soft- and mineralized-tissues substantially differ in their healing processes and outcomes (3).

The bone healing process occurs throughout orchestrated and overlapping phases, starting with a transient inflammatory response that constructively influences subsequent events such as angiogenesis and fibrous connective tissue formation, osteogenic cellular differentiation, and bone formation (6–9). Along bone healing, macrophages are described to be present mainly in the inflammatory phase and are regarded as an important source of pro-inflammatory cytokines (3, 10), which theoretically amplify the recruitment of its own lineage and other immune cells. Indeed, activated macrophages can release multiple mediators, including pro-inflammatory and anti-inflammatory cytokines, and growth factors (11). In this context, the early release of cytokines and growth factors in bone injury sites is associated with a positive intramembranous and endochondral healing outcome (12–14). Moreover, studies suggest that macrophages contribution to bone healing extends beyond earlier inflammatory events and can include the production of growth factors that direct mesenchymal stem cells (MSCs) into osteogenic differentiation (11). Accordingly, macrophages depletion in mice significantly suppresses woven bone deposition and mineralization during bone fracture healing in endochondral bones (15).

It is mandatory to consider that *in vivo* studies concerning macrophages functions in bone healing are predominantly focused on endochondral bone healing following fractures (3, 6, 16). Importantly, endochondral and intramembranous bones have distinctive features, which include substantial differences on healing mechanisms (17). Indeed, while long bone heals *via* endochondral ossification, intramembranous bones (such maxillary alveolar dental socket) healing takes place without cartilage formation (3). Also, while bone fracture sites are usually a sterile milieu, oral tissues surrounding the alveolar bone are under a constant microbial challenge (18). Consequently, the scarce information about macrophage contributions on endochondral bone healing cannot be directly translated to intramembranous bone healing, where its role remains to be determined.

In this context, we previously characterized an alveolar bone healing model after tooth extraction in C57Bl/6 mice, allowing further investigations concerning the role of the immunological system components on intramembranous bone healing (7, 12). Interestingly, a series of macrophage-related growth factors and cytokines, and members of the chemokine family with a potential role in macrophage chemoattraction were found to be upregulated during alveolar bone healing process (7). Specifically, upregulation of the chemokine receptor CCR2, chemokine (C-C motif) receptor 2, and its cognate chemokine (C-C motif) ligand 2 (CCL2) suggests a role for CCR2/CCL2 axis in macrophages migration to bone injury sites (7). Indeed, the chemokine system recruits different leukocytes subsets into the specific microenvironments, where chemokines bind to their respective receptors selectively expressed by each leukocyte subset (19). Exemplifying, while resident macrophages are characterized by the expression of CX3CR1, chemokine receptors CCR2 and CCR5 are expressed by inflammatory monocytes/macrophages and mediate its traffic into injured tissues in response to chemokines such as CCL1, CCL2, and CCL5 (19). CCR2 has been regarded a key player regulating the macrophages influx into injured tissues throughout tissue healing (2, 10). Indeed, CCR2-deficient mice have an impaired recruitment of F4/80+ cells (suggested as macrophages) on different sites of injury (2, 20), including endochondral bones (3, 10), which consequently delays the evolution of the subsequent healing (2). In addition, previous studies demonstrate the F4/80 and CCR2 co-expression in murine macrophages (2), and the association of CCR2 with the migration of F4/80+ cells from blood into inflamed and healing sites (2, 3, 10, 20, 21). Indeed, the majority of F4/80+ cells recruited from blood into inflamed sites are monocytes/macrophages, which co-express CCR2 (2), being the CCR2 targeted disruption associated with decreased F4/80+ cells number of these in injuries sites (2, 3, 10, 20, 21). However, the molecular and cellular mechanisms triggered by CCR2+ cell migration and its impact in the intramembranous bone healing remain unaddressed.

Taking into consideration that bone healing depends of an initial and transient inflammatory response, and that macrophages are key regulators of this process (22), it is reasonable to hypothesize that CCR2 deficiency can reduce F4/80+ cells migration and negatively affect the intramembranous bone healing. Therefore, we investigated the role of CCR2 on F4/80+ cells migration to bone healing sites and its consequences to the subsequent intramembranous bone healing outcome, by means of the CCR2KO and C57Bl/6-wild-type (WT) mice strains comparative analysis using micro-computed tomography (μCT), histological, immunological, and molecular methods.

## Materials and Methods

### Animals

The experimental groups were comprised of 8-week-old male WT C57BL/6 mice and mice with targeted disruption of the CCR2 (CCR2KO, C57BL/6 background), both WT and CCR2KO littermates bred in the animal facilities of USP. Mice were fed with sterile standard solid mice chow (Nuvital, Curitiba, PR, Brazil) and sterile water throughout the study period, except on the first 24 h after surgery, in which diet was crumbled. The experimental groups comprised nine mice (five animals for microscopic analysis and four animals for the PCR array analysis). This study was carried out in strict accordance with the recommendations in the Guide for the Care and Use of Laboratory Animals of the National Institutes of Health. The experimental protocol was approved by the local Institutional Committee for Animal Care and Use (Committee on Animal Research and Ethics CEEPA-FOB/USP, process #005/2012).

### Mice Tooth Extraction Model

The surgical procedures for tooth extraction were performed as described (7). In brief, the animals received general anesthesia by intramuscular administration 80 mg/kg of ketamine chloride (Dopalen, Agribrans do Brasil LTDA) and 160 mg/kg of xylazine chloride (Anasedan, Agribrands do Brasil LTDA) in the proportion 1:1. The upper right incisor was extracted with a dental probe, as previously described (7). After 0 h, 7, 14, and 21 days post tooth extraction, mice were euthanazied and maxillae were harvested. Maxillae for the μCT and histological analyses were fixed in PBS-buffered formalin (10%) solution (pH 7.4) for 48 h at room temperature, subsequently washed overnight in running water and maintained temporarily in alcohol fixative (70% hydrous ethanol) until the conclusion of the μCT analysis, and then decalcified in 4.13% EDTA (pH 7.2) and submitted to histological processing. Samples from maxillae containing only the region of the alveolus were destined to molecular analysis were stored in RNAlater (Ambion, Austin, TX, USA) solutions (7).

### μCT Analysis

The maxillae at 0 h, 7, 14, and 21 days post tooth extraction were scanned by the Skyscan 1174 System (Skyscan, Kontich, Belgium) at 50 kV, 800 µA, with a 0.5 mm aluminum filter and 15% beam hardening correction and 180 ° of rotation. Images were captured with a resolution of 14 µm pixel size and reconstructed using the NRecon software. Three-dimensional (3D) images were rendered using CTVox software, and quantitative parameters were assessed using CTAn software following recommended guidelines and a previously μCT characterization (7, 23, 24). Newly formed bone was segmented in a cylindrical region of interest (ROI) covering the entire length of the alveolus (3 mm) and a diameter of 1 mm. The following morphological parameters were assessed: bone volume fraction [Bone Volume/Tissue Volume (BV/TV), %], trabecular thickness (Tb.Th, mm), trabecular number (Tb.N, mm), and trabecular separation (Tb.Sp) (24).

### Histology Sample Preparation and Histomorphometric Analysis

After μCT scanning, maxillae were immersed in buffered 4% EDTA for demineralization and processing for embedding in paraffin blocks. Transversal serial 5-µm slices from medial third were cut for histology with H&E staining, picrosirius red, immunohistochemistry, and immunofluorescence. A total of three histological sections from central region of the alveolar socket stained by H&E were used to quantify the following healing components: clot formation, inflammatory infiltrate, connective tissue (collagen fibers, fibroblasts, and blood vessels), bone matrix, osteoblasts, osteoclasts, and other components (empty spaces and bone marrow), as previously described (7). The identification and quantification of healing components was performed by a single calibrated investigator with a binocular light microscope (Olympus Optical Co., Tokyo, Japan) using a 100× immersion objective and a Zeiss kpl 8× eyepiece containing a Zeiss II integration grid (Carl Zeiss Jena GmbH, Jena, Germany) with 100 points in a quadrangular area. The grid image was successively superimposed on 13 histological regions per histological section, totaling 3 sections for each specimen. Only the points coincident with the histological components were considered, and the total number of points was obtained to calculate the area density for each healing component in each section.

### Birefringence Analysis

Birefringence analysis was performed with picrosirius-polarization method, to identify and quantify collagen content, as well compare the quality of bone trabeculae matrix as previously described (7). Briefly, four histological from central region of the each alveolar socket were stained with Picrosirius Red Stain, and the images were captured by a polarizing lens coupled to a binocular inverted microscope (Leica DM IRB/E) using a 10× objective. Adobe Photoshop CS6 software was used to delimit the ROI, the socket area filled with new tissue, as well to exclude bony edges of the alveolar margins or residual old bone. The quantification of the intensity of birefringence brightness (pixels^2^) was performed using the AxioVision 4.8 software (CarlZeiss) to define total area of green, yellow, and red collagen fibers.

### Immunohistochemistry and Immunofluorescence

Histological sections from 0, 7, 14, and 21 days were deparaffinized following standard procedures. For immunohistochemistry, the material was pre-incubated with 3% Hydrogen Peroxidase Block (Spring Bioscience Corporation, CA, USA) and subsequently incubated with 7% NFDM to block serum proteins. The histological sections from both, WT and CCR2 KO mice, were then incubated with anti-CCR2 polyclonal primary antibody (Santa Cruz, #sc-31564), anti-F4/80 (a pan macrophage marker for mice) polyclonal primary antibody (Santa Cruz, #sc-26642), anti-CD68 polyclonal primary antibody (Santa Cruz, #sc-7084), anti-CCR5 polyclonal (Santa Cruz, #sc-6129) at 1:100 concentrations, and with anti-Ly6g-Gr1 polyclonal antibody (Santa Cruz, #sc-168490) and anti-CD3 polyclonal antibody (Santa Cruz, #sc-1127) at 1:50 concentrations, anti-CCR5 polyclonal (Santa Cruz, #sc-6129) at 1:100 concentrations, for 1 h at room temperature. Universal immuno-enzyme polymer method was used, and sections were incubated in immunohistochemical staining reagent for 30 min at room temperature. The identification of antigen–antibody reaction was performed using 3,3′-diaminobenzidine and counterstaining with Mayer’s hematoxylin. For control staining of the antibodies, serial sections were treated only with the Universal immuno-enzyme polymer, in a separate preparation. For immunofluorescence, sections from WT at 7 days were rehydrated and retrieved the antigens by boiling the histological slides in 10 mM sodium citrate buffer pH 6 for 30 min at 300°C. Subsequently, the sections were permeabilized with 0.5% Triton X-100 in PBS and blocked with blocking solution (1% bovine serum albumin diluted in 1× PBS), for 1 h at room temperature. For immunolocalization of CCR2+ CCR5+ macrophages, the sections were incubated with both primary antibodies: anti-CCR2 rabbit monoclonal antimouse (Abcam, #ab203128) and anti-CCR5 goat polyclonal antimouse (Santa Cruz, #sc-6129). All primary antibodies were diluted at 1:100 in blocking solution and incubated over night at 4°C. After repeated washing steps with PBS (3 times, 10 min each wash), the sections were incubated with both secondary antibodies: Alexa Fluor555 goat anti-rabbit secondary antibody (Life Technologies, #A21428) and Alexa Fluor488 donkey anti-goat (Life Technologies, #A11055), diluted at 1:150 in blocking solution, incubated for 2 h at room temperature. Sections were nuclear stained with DAPI (Thermo Fisher Scientific, #D3571) diluted at 3 µM in ddH_2_O for 10 min, mounted in with ProLong Gold Antifade Reagent (Invitrogen, #P36930). Imaging was performed in a Nikon Eclipse Ni-U upright fluorescence microscope (Nikon instruments) equipped with a Zyla 5.5 sCMOS camera (Andor).

#### Quantification of Immunolabeled Cells

The analysis of immunolabeled cells was performed by a single calibrated investigator with a binocular light microscope (Olympus Optical Co., Tokyo, Japan) using a 100× immersion objective, following the similar criteria described previously for histomorphometric analysis in H&E (see Histology Sample Preparation and Histomorphometric Analysis). Briefly, five samples (biological replicate) for each experimental period and strains were used for quantitative analysis. A total of three sections of each sample (technical replicate) containing the central region of the alveolar socket was used to quantify immunolabeled cells for each mentioned target (F4/80, CCR2, CCR5, Ly6g-Gr1, CD3, and CD68). A total of 13 fields (100 points in a quadrangular area) were analyzed using Zeiss II integration grid (100 points) (Carl Zeiss Jena GmbH, Jena, Germany) for each section. Only the points coincident with the immunolabeled cells were considered in cell counting, and the mean for each section was obtained for statistical analysis.

### Isolation of F4/80+ Cells From Alveolar Socket and Flow Cytometric Analysis

The isolation and characterization of monocytes/macrophages from the alveolar socket at day 7 post tooth extraction was performed as previously described (25). The alveolar socket tissues from five C57Bl/6 mice were collected at day 7 post tooth extraction, and subsequently were fragmented, weighed, and incubated for 1 h at 37°C, in RPMI-1640 supplemented with NaHCO_3_, penicillin/streptomycin/gentamycin and liberate blendzyme CI (Roche-F. Hoffmann-La Roche Ltd., Basel, Switzerland). The samples were processed in the presence of 0.05% DNase (Sigma-Aldrich, Steinhein, Germany) using Medimachine (BD Biosciences Pharmingen, San Diego, CA, USA), according to the manufacturer’s instructions. The cell viability was assessed by Trypan blue exclusion assay, and the cell count was performed in a hemocytometer, with these data depicted in the manuscript as the total monocyte/macrophage cell count. For flow cytometry analysis, after counting the cells were stained for 20 min at 4°C with the optimal dilution of each antibody; phycoerythrin- and fluorescein isothiocyanate-conjugated antibodies against CCR2, CCR5, and F4/80-anti mouse antibodies, as well with respective isotype controls (BD Biosciences Pharmingen, San Diego, CA, USA), and analyzed by FACScan and CellQuest software (BD Biosciences Pharmingen, San Diego, CA, USA). Results are presented as the number of F4/80+ CCR2+ cells and F4/80+ CCR5+ cells ± SD in the alveolar socket of each mouse.

### RealTime PCR Array Reactions

RealTime PCR array reactions were performed as previously described (7). Only hemimaxillae containing the region of the alveolus socket were used as experimental samples, while the hemimaxillae without injury were used as tissue control. Samples were storage in RNA Stabilization Solution (RNAlater^®^, Thermo Fisher Scientific, Waltham, MA, USA) until RealTime PCR array reactions. The extraction of total RNA from remaining alveolus with 0 h, 7, 14, and 21 days post-extraction from WT and CCR2KO was performed with RNeasyFFPE kit (Qiagen Inc., Valencia, CA, USA) according to the manufacturers’ instructions. First, RealTime PCR array was performed from a pool of all experimental time points (0 h, 7, 14, and 21 days), providing targets in which expression variation presented a significant variation compared with the control side. Then, upregulated targets were analyzed regarding their kinetics of expression for specific time points of 0, 7, 14, and 21 days throughout the alveolar bone healing. The integrity of the RNA samples was verified by analyzing 1 µg of total RNA in a 2100Bioanalyzer (Agilent Technologies, Santa Clara, CA, USA) according to the manufacturers’ instructions, and the complementary DNA was synthesized using 3 µg of RNA through a reverse transcription reaction (Superscript III, Invitrogen Corporation, Carlsbad, CA, USA). RealTime PCR array was performed in a Viia7 instrument (Life Technologies, Carlsbad, CA, USA) using a custom panel containing targets “Wound Healing” (PAMM-121), “Inflammatory cytokines and receptors” (PAMM-011), and “Osteogenesis” (PAMM-026) (SABiosciences, Frederick, MD, USA) for gene expression profiling. RealTime PCR array data were analyzed by the RT^2^ profiler PCR Array Data Analysis online software (SABiosciences, Frederick, MD, USA) for normalizing the initial geometric mean of three constitutive genes (GAPDH, ACTB, and Hprt1) and subsequently normalized by the control group, and expressed as fold change relative to the control group, as previously described (26, 27). Data are expressed as heat map fold change relative to the control group.

### Statistical Analysis

Differences among data sets were statistically analyzed by one-way analysis of variance followed by the Tukey multiple comparison post test or Student’s *t*-test where applicable. For data that did not fit in the distribution of normality, the Mann–Whitney and Kruskal–Wallis (followed by the Dunn’s test) tests were used. The statistical significance of the experiment involving the PCR Array was evaluated by the Mann–Whitney test, and the values tested for correction by the Benjamini–Hochberg procedure (28). Values of *p* < 0.05 were considered statistically significant. All statistical tests were performed using the GraphPad Prism 5.0 software (GraphPad Software Inc., San Diego, CA, USA).

## Results

### Immunohistochemistry of Inflammatory Infiltrate Throughout Intramembranous Bone Healing in Mice

**Figure 1:**
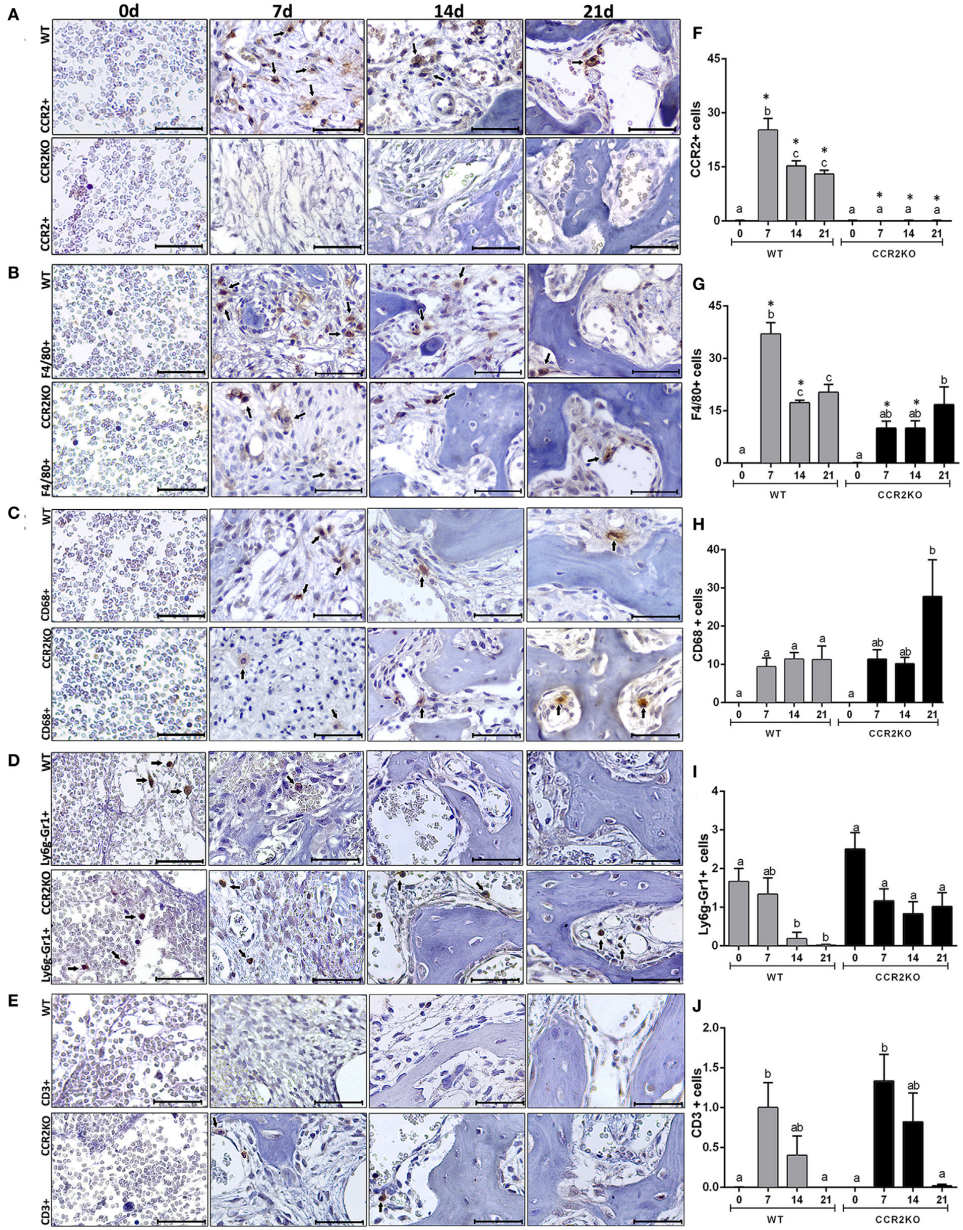
Immunolabeling of inflammatory infiltrate in the intramembranous alveolar bone healing in mice. Representative sections from medial thirds of the socket at 0, 7, 14, and 21 days post tooth extraction with immunolabeling of CCR2+ cells **(A)**, F4/80+ cells **(B)**, CD68+ cells **(C)**, Ly6g-Gr1+ cells **(D)**, and CD3+ cells **(E)** in wild-type (WT) **(A)** and CCR2KO mice **(B)**, as indicated with arrows (scale bar = 100 µm). Quantitative and comparative analysis of CCR2+ cells **(F)**, F4/80+ cells **(G)**, CD68+ cells **(H)**, Ly6g-Gr1+ cells **(I)**, and CD3+ cells **(J)** in WT vs CCR2KO mice at days 0, 7, 14, and 21 post-extraction. Different letters indicate significant differences in each time point (*p* < 0.05); symbol * indicate significant differences between WT vs CCR2KO at the same time point.

In the view of the primary role of CCR2 in the migration of monocytes/macrophages into sites of inflammation (29), we used immunohistochemistry to address the presence CCR2+ cells, F4/80+ and CD68+ cells (macrophages), Ly6g-Gr1+ cells (polymorphonuclear leukocyte/neutrophils), and CD3+ cells (lymphocytes) on the site of alveolar bone healing at different time points (0, 7, 14, and 21 days) post tooth extraction in C57Bl/6 WT mice an CCR2KO mice (Figures 1A–J). At 0 day time point, there was a peak of Ly6g-Gr1+ cells (Figure 1I) observed in the blood clot formed post-extraction in WT and CCR2KO mice, with a significant decrease from 7 to 21 days in C57Bl/6 mice, and with no differences observed among different time points in CCR2KO mice. During the early inflammatory phase (7 days), there was a peak of area density for CCR2+ (Figure 1F) and F4/80+ (Figure 1G) cells in the granulation tissue and inflammatory infiltrate in the socket of C57BL/6 mice. At 14 days, CCR2+, F4/80+, and CD68+ cells were found in permeating the connective tissue surrounding bone formation areas, while at 21 days, these cells were found predominantly in the bone marrow and surrounding blood vessels (Figures 1A–C). The influx of F4/80+ cells was significantly reduced at 7 and 14 days in CCR2KO compared with WT mice (*p* < 0.05) (Figures 1E,F). The number of both type of cells, CCR2+ and F4/80+ cells, was significantly decreased (*p* < 0.05) at 14 and 21 days compared with 7 days in C57Bl/6 WT mice (Figures 1B–D). No significant differences were observed for CD3+ cells in the WT vs CCR2KO comparisons (Figure 1). A different kinetics for Ly6g-Gr1+ and CD68+ cells infiltration was observed in CCR2KO mice compared with WT, with a slight higher number of Ly6g-Gr1+ and CD68+ cells in CCR2KO mice compared with WT at 21 days. However, no significant differences were found between WT vs CCR2KO comparisons in specific time points (Figures 1H,I).

### μCT Analyses of Intramembranous Bone Healing in WT vs CCR2KO

**Figure 2:**
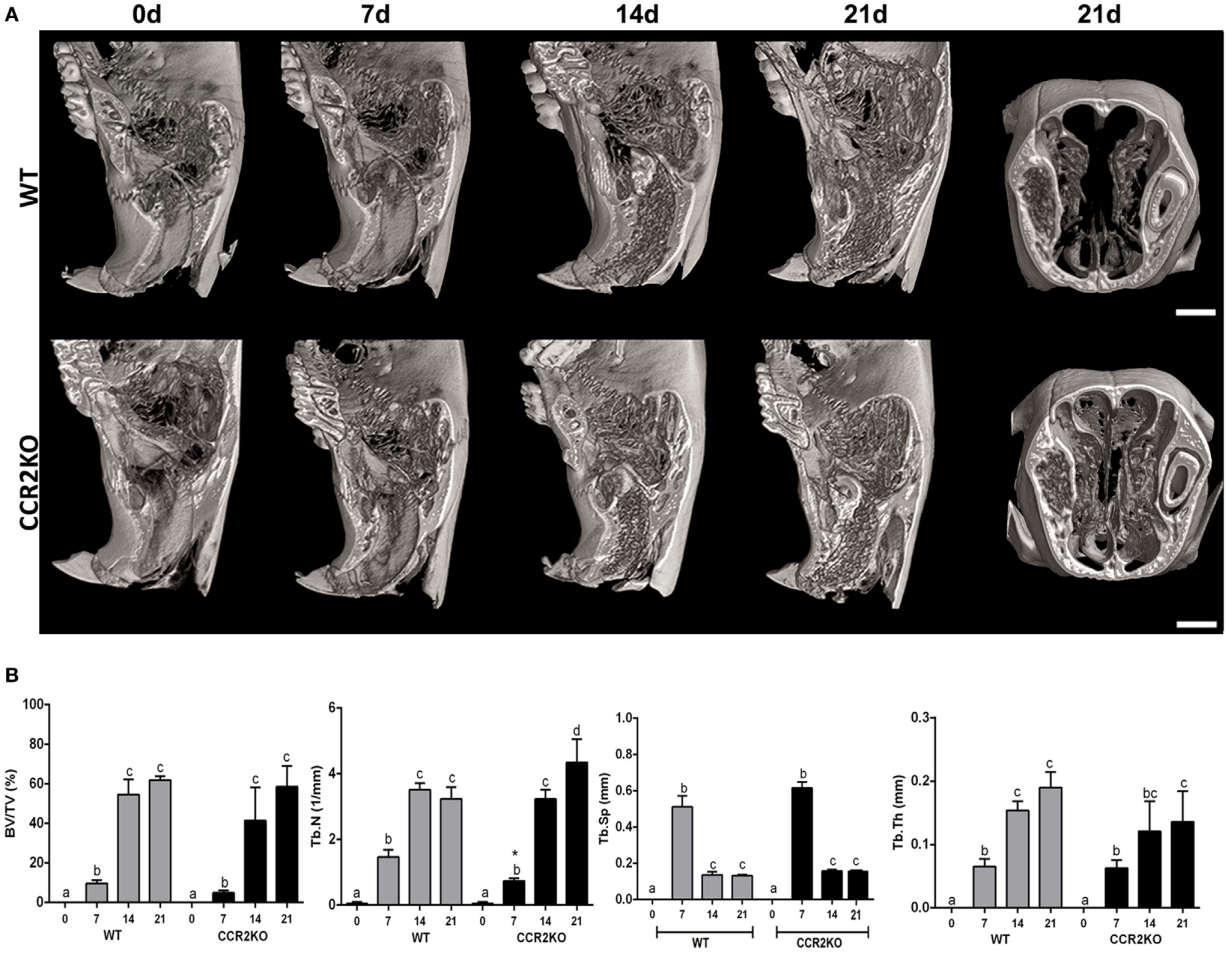
Micro-computed tomography (μCT) analysis along intramembranous bone healing in wild-type (WT) vs CCR2KO mice. Maxillae containing dental sockets post-extraction from WT and CCR2KO mice were scanned with the μCT System (Skyscan 1174; Skyscan, Kontich, Belgium). **(A)** Three-dimensional images obtained by CT-Vox software reveal the socket morphology at 0 h, 7, 14, and 21 days post tooth extraction. **(B)** Morphological parameters such as bone volume fraction (BV/TV, %), trabecular number (Tb.N, 1/mm), trabecular separation (Tb.Sp, mm), and trabecular thickness (Tb.Th, mm) were quantified at 7, 14, and 21 days post tooth extraction. Results are presented as mean and SD to different parameters. Different letters indicate significant differences in each time point (*p* < 0.05); symbol * indicate significant differences between WT vs CCR2KO at the same time point.

The sagittal 3D images of maxillae containing socket area (Figure 2A) and quantitative assessment of the bone morphological parameters from μCT analysis (Figure 2B) did not indicate major differences in the inorganic bone matrix between WT and CCR2KO mice. At the 0 day time point, both WT and CCR2KO mice presented sockets with an absence of hyperdense areas. At 7 days, negligible hyperdense areas were observed from the lateral and apical walls of the extraction sockets, while at 14 and 21 days, hyperdense structures compatible with new trabecular bone were observed filling the entire socket in both, WT and CCR2KO mice (Figure 2A). In general, the values of morphological parameters such as volume fraction (BV/TV), trabecular thickness (Tb.Th), and trabecular number (Tb.N) were progressively increased from 7 to 21 days (*p* < 0.05), while the trabecular separation (Tb.Sp) were inversely reduced (*p* < 0.05) in both, WT and CCR2KO mice. Specifically, at 7 days, the Tb.N parameter was significantly lower in the CCR2KO compared with WT mice (Figure 2B).

### Histological and Birefringence Analysis of Healing Components in WT vs CCR2KO During the Alveolar Bone Healing

**Figure 3:**
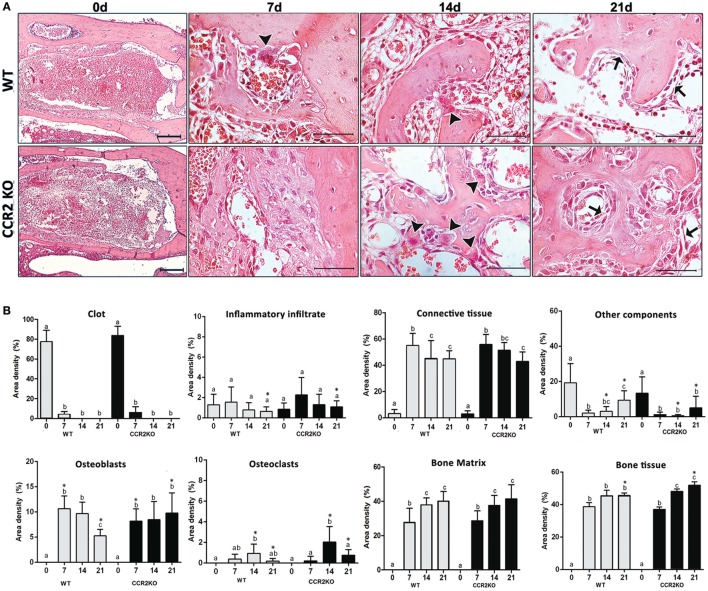
Histological and histomorfometric analysis of healing components during the intramembranous bone healing in wild-type (WT) vs CCR2KO mice. **(A)** Comparative morphology of the healing phases at 0 h, 7, 14, and 21 days post-extraction of upper right incisor, stained with H&E (0 h—10× magnification and bar = 200 µm; 7, 14, and 21 days—40× magnification and bar = 100 µm). **(B)** Results are presented as the mean of area density for each structure measured in each examined group. Different letters indicate significant differences in each time point (*p* < 0.05); symbol * indicates significant differences between WT vs CCR2KO at the same time point (arrowheads = osteoclasts; arrows = osteoblasts).

**Figure 4:**
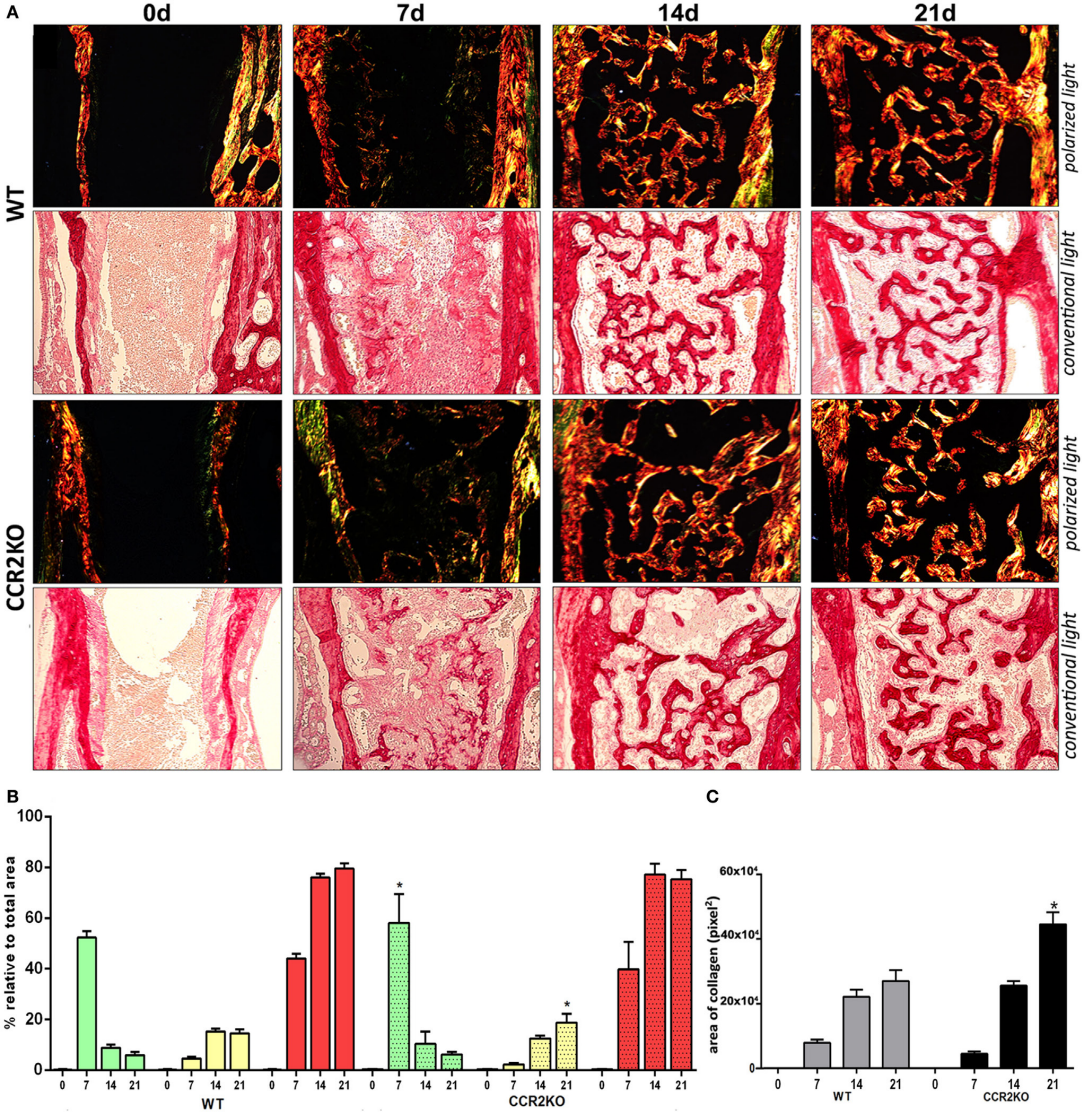
Birefringence analysis of collagen fiber bundles maturation along intramembranous bone healing in wild-type (WT) vs CCR2KO mice. **(A)** Representative sections from medial thirds of the socket stained with Picrosirius red upon polarized and conventional light at 0 h and 7, 14, and 21 days (20× magnification). Green birefringence color indicates thin fibers, while yellow and red colors indicate thick collagen fibers. **(B)** Intensity of birefringence performed using image-analysis software (AxioVision, v. 4.8, CarlZeiss) from each color (%) to quantify thin and thick collagen fibers, as well **(C)** total area of collagen fibers (pixels^2^) in WT vs CCR2KO mice. Results are presented as mean and SD of % **(B)** and pixels^2^ **(C)**. Symbol * indicates a statistically significant difference (*p* < 0.05) between WT vs CCR2KO at the same time point.

Histological analysis demonstrated that the intramembranous bone healing process followed suitable overlapping phases in both strains (WT and CCR2KO), although minor morphological and quantitative differences were observed between them at specific time points (Figures 3A,B). Overall, the socket of both (WT and CCR2KO mice) exhibited predominantly blood clot at day 0 (immediately after tooth extraction) with a negligible number of leukocytes. Subsequently, were observed an abundant amount of granulation tissue (blood vessels, fibers from connective tissue, and fibroblasts) with leukocytes infiltration at 7 days, as well as bone formation from remaining bone edges. At 14 days, an intense bone remodeling activity was evidenced by the presence of osteoclasts, while organized matrix surrounding blood vessels and bone marrow were present at 21 days (Figure 4A). Comparatively, the absence of CCR2 resulted in an increased area density (%) of fibroblasts at 7 days; blood vessels at 14 days; osteoclasts at 14 and 21 days, osteoblasts; fibers from connective tissue and inflammatory infiltrate at 21 days; in CCR2KO vs WT (*p* < 0.05). On the other hand, CCR2KO showed a reduced area density of osteoblasts at 7 days, fibroblasts, fibers from connective tissue at 14 days; and other components (especially bone marrow) at 21 days (*p* < 0.05; WT vs CCR2KO) (Figure 4B). In the birefringence analysis, the new organic matrix consisting predominantly of collagen fibers bundles were found from 7 to 21 days inside the socket in both WT and CCR2KO mice, as evidenced by images under polarized light (Figure 4A). The quantitative analysis showed a similar pattern in the matrix maturation dynamics during the time points in both WT and CCR2KO mice (Figure 4B). While the area of collagen fibers in green tones (thinner and immature fibers) significantly decreased from 7 to 21 days, collagen fibers emitting yellow and red color spectrum (thicker and mature collagen) increased at these same time points (*p* < 0.05). Of note, CCR2KO mice showed an increased quantity of green fibers (7 days) and yellow fibers (21 days) compared with WT mice (*p* < 0.05). Also, the total amount of collagen fibers bundles (sum of color spectrums) was significantly increased in CCR2KO mice at 21 days (*p* < 0.05 vs WT) (Figure 4C).

### Differential Gene Expression Between WT vs CCR2KO During the Alveolar Bone Healing

**Figure 5:**
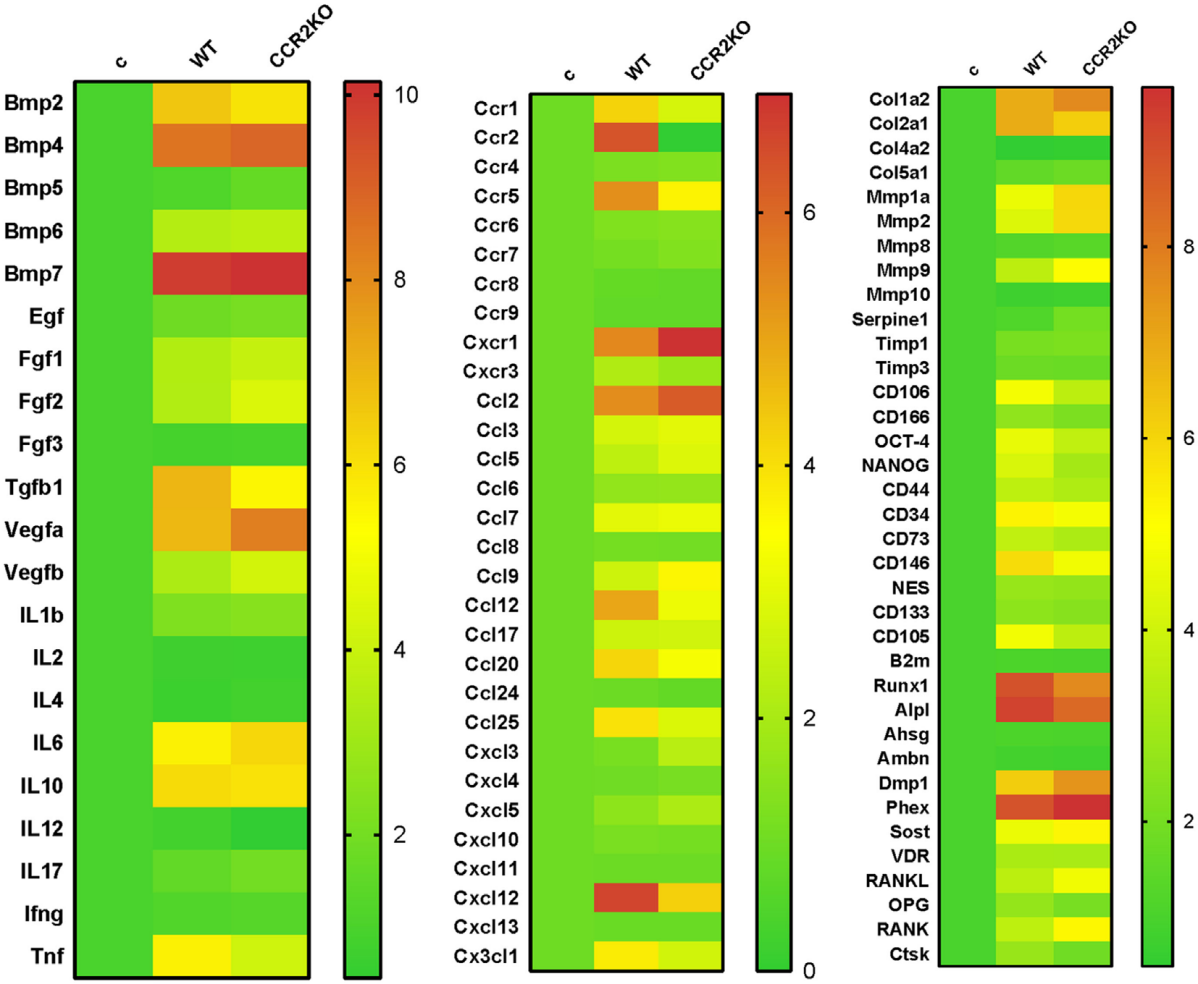
Gene expression patterns in the intramembranous bone healing after tooth extraction in wild-type (WT) vs CCR2KO mice. Molecular analysis of the gene expression patterns in the bone healing was performed with a pool of samples from all the experimental time periods (0, 7, 14, and 21 days) for growth factors, cytokines, chemokines, and chemokine receptors, ECM/repair markers, mesenchymal stem cells, and bone markers. Gene expression was performed by using exploratory analysis by RealTime PCR array, with the VIA7 system (Applied Biosystems, Warrington, UK) and a customized qPCR array comprised of the major targets (Osteogenesis, Inflammatory Cytokines & Receptors, and Wound Healing panels) of the PCR array RT^2^ Profiler (SABiosciences/QIAGEN). RealTime PCR array analysis was performed with the VIA7 system (Applied Biosystems, Warrington, UK) using a customized qPCR array comprised of the major targets from the Osteogenesis, Inflammatory Cytokines & Receptors, and Wound Healing panels of the PCR array RT^2^ Profiler (SABiosciences/QIAGEN). Results are depicted as the fold increase change (and the SD) in mRNA expression from triplicate measurements in relation to the control samples and normalized by internal housekeeping genes (GAPDH, HPRT, and β-actin).

**Figure 6:**
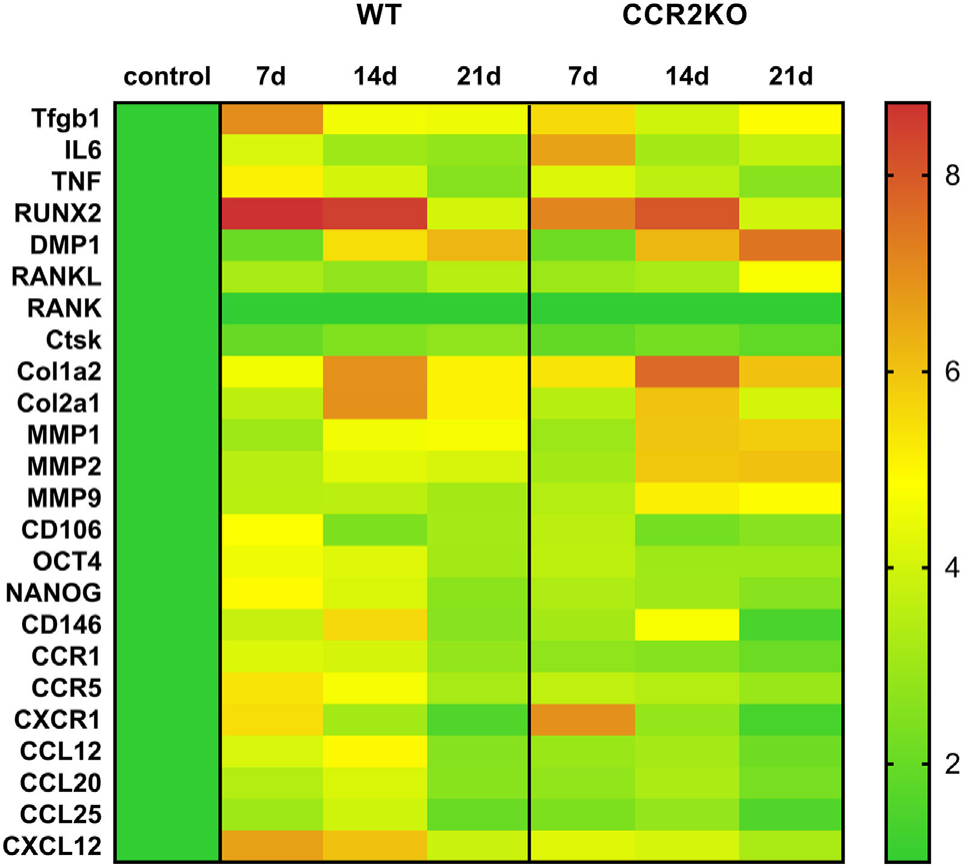
Kinetics of gene expression in the intramembranous bone healing after tooth extraction in wild-type (WT) vs CCR2KO mice. RealTime PCR array pooled from of all the experimental time periods for each strain (WT and CCR2KO mice) was used to identify targets with a significant expression variation for their subsequent analyses in different time points along bone healing post tooth extraction (7, 14, and 21 days). RealTime PCR array analysis was performed with the VIA7 system (Applied Biosystems, Warrington, UK) using a customized qPCR array comprised of the major targets from the Osteogenesis, Inflammatory Cytokines & Receptors, and Wound Healing panels of the PCR array RT^2^ Profiler (SABiosciences/QIAGEN). Results are depicted as the fold increase change (and the SD) in mRNA expression from triplicate measurements in relation to the control samples and normalized by internal housekeeping genes (GAPDH, HPRT, and β-actin).

Differential gene expression of several molecules involved in bone healing (i.e., growth factors, bone formation markers, immunological markers, and putative MSC markers) was investigated in CCR2KO and WT strains. We performed an exploratory analysis by RealTime PCR array with a pool from samples of all time points in both WT and CCR2KO mice (Figure 5) followed by kinetics of expression analysis for selected targets (Figure 6). Of note, the mRNA expression of growth factor TGFB1 and putative MSC markers (CD106, OCT4, NANOG, and CD146) was upregulated in WT mice, with a peak of mRNA levels at 7 days, while those same targets were significantly decreased in CCR2KO at the same time point (*p* < 0.05). Among the bone markers evaluated, the mRNA expression of the early bone formation marker RUNX2 was also significantly reduced in CCR2KO compared with WT mice (*p* < 0.05) at 7 and 14 days, while RANKL was significantly increased in CCR2KO at 21 days (Figure 6). For ECM markers, the mRNA levels of Col1a2 and MMP1, MMP2, and MMP9 were increased in CCR2KO mice at 14 and 21 days. Considering immunological markers (cytokines, chemokines, and its receptors), while CCR5 and TNF mRNA levels were decreased in CCR2KO compared with WT, CXCR1 and IL6 were increased in the pooled samples analysis (Figure 5) and at 7 days (Figure 6). In the kinetics of expression, the TNF, CXCR1, and IL6 mRNA levels peaked at day 7, with a higher expression of TNF in WT mice and a higher expression of CXCR1 and IL6 in CCR2KO mice (*p* < 0.05).

### Immunological Analysis of Macrophages Along Intramembranous Bone Healing in WT vs CCR2KO Mice

**Figure 7:**
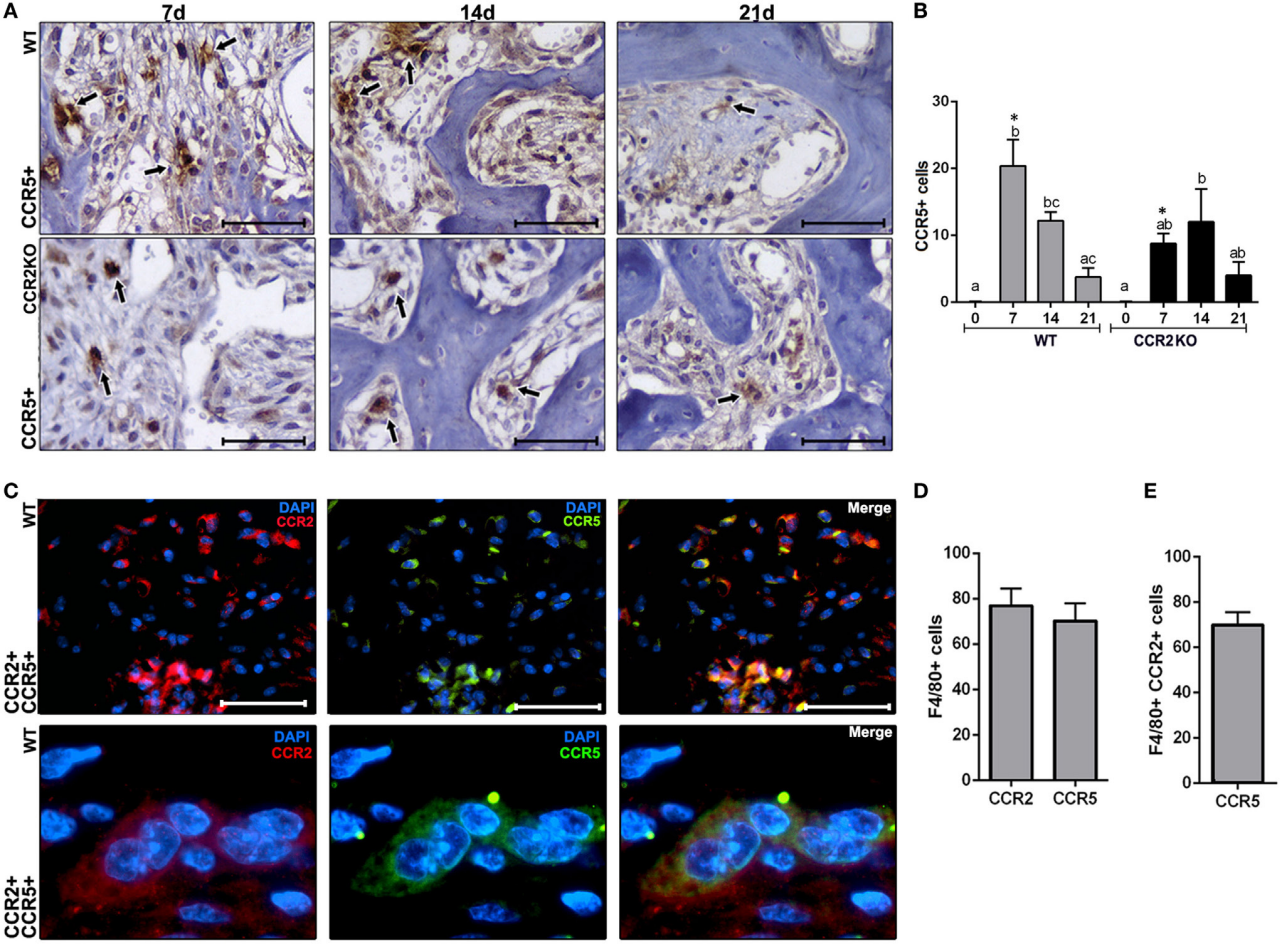
Immunological analysis of CCR2 and CCR5 along intramembranous bone healing in wild-type (WT) vs CCR2KO mice. **(A)** Representative sections from medial thirds of the socket at days 7, 14, and 21 post tooth extraction with immunolabeling for CCR5+ cells in WT and CCR2KO mice (as indicated with arrows) (40× magnification; scale bar = 100 µm). **(B)** Quantitative analysis of CCR5+ cells in WT and CCR2KO mice at days 0, 7, 14, and 21 post-extraction. Different letters indicate significant differences in each time point (*p* < 0.05); symbol * indicate significant differences between WT vs CCR2KO at the same time point. **(C)** Immunocolocalization of CCR2 (TRITC) and CCR5 (fluorescein isothiocyanate) in inflammatory cells at day 7 post tooth extraction in WT mice (100× magnification). **(D,E)** The phenotype of F4/80+ cells from alveolar socket at day 7 post tooth extraction in WT mice, evaluated by flow cytometry, and depicted as the number. Results are presented as mean and SD.

We used immunohistochemistry to identify and compare the number CCR5+ cells in WT and CCR2KO during alveolar bone healing at different time points (0, 7, 14, and 21 days), as well immunofluorescence and flow cytometry to identify CCR2+ CCR5+ cells in WT mice. CCR5+ cells are present throughout the alveolar bone healing in both, WT and CCR2KO mice (Figure 7A). However, there was a peak of CCR5+ cells at 7 days in WT mice, while CCR2KO demonstrated a significantly reduced number of these cells (*p* < 0.05) (Figure 7B). As evidenced by immunofluorescence at 7 days, CCR2 and CCR5 are co-localized in cell with a suggestive monocyte/macrophage morphology during the alveolar bone healing in C57Bl/6 mice (Figure 7C). FACS analyses of F4/80+ cells from alveolar bone healing at 7 days post-extraction in WT mice revealed that 70% of F4/80+ CCR2+ macrophages are also positive for CCR5 (Figures 7D,E).

## Discussion

Macrophages are among the first immune cells required to trigger and modulate the inflammatory response, and their initial recruitment from circulation into injured tissues is an essential initial event for a proper tissue healing (1–4, 28). In this context, inflammatory macrophages subpopulation is characterized by high levels of CCR2 expression (21, 28–30), and CCR2 and its ligand CCL2 are upregulated along alveolar bone healing (7), suggesting its involvement in macrophages migration throughout the healing events. At this point, contributions of CCR2 in mediating recruitment of blood/medullar monocytes to inflamed tissues has been demonstrated in different models of injury (2, 3, 10), but its specific function on craniofacial bone is repair still unclear (7). Therefore, in this study, we performed a comparative characterization of the alveolar bone healing process in CCR2KO and C57Bl/6-WT mice, to investigate the role of CCR2 in intramembranous bone healing post tooth extraction.

As previously demonstrated by other models of injury in mice, the majority of F4/80+ cells recruited from blood into inflamed sites are monocytes/macrophages, which also exhibit CD11b and CCR2 expression (2). Consequently, the targeted disruption of the CCR2 in CCR2KO mice significantly decreased the number of these F4/80+ cells in cutaneous wounds (2), muscle (20), and endochondral bone injuries (3, 10, 21). In this context, despite the complexity of macrophages phenotype, F4/80 has been considered as pan marker for murine macrophages in these specific models of injury. Accordingly, our results initially demonstrate the presence of CCR2+ and F4/80+ cells in the bone healing sites in C57Bl/6 mice, with a peak at inflammatory stage. Interestingly, while F4/80+ cells influx was significantly reduced in CCR2KO mice, suggesting in a cause-and-effect manner the contribution of CCR2 to macrophages migration.

We next investigate if the negative impact of CCR2 deficiency on F4/80+ cells migration was translated into modifications of the subsequent bone healing stages (Figures 2–4). The μCT analysis demonstrated that absence of CCR2 did not resulted in major changes in the mineralization pattern and bone microarchitecture along alveolar healing in CCR2KO strain (Figure 2). Bone mineralization was detected from 7 days in alveolar socket and was gradually increased until the endpoint period (21 days), with important changes on morphological parameters (Tb.Th, Th.Sp, and Th.N) (7), when the alveolar socket is filled whit a thick bone trabeculae and well-defined medullary canals, being such kinetics in accordance with other experimental models in rodents (7, 15, 31). In addition, the histological features of bone healing observed in this study are in accordance to previous description for intramembranous bone healing in mice (7) as well as in humans (32). As well, the birefringence analysis demonstrates a similar evolution in the maturation, organization, and arrangement of collagen fibers inside the alveolar socket in both CCR2KO and WT strains, in accordance with a previous description (7). Taking the μCT and microscopic data together, it is evident that CCR2 deficiency do not impair alveolar bone healing, which is in opposition to endochondral bone healing (6, 10), where CCR2KO mice show a delayed bone formation and maturation during healing. However, as previously mentioned, endochondral and intramembranous bones have fundamental embryological, anatomical, and functional differences (7, 17). Despite of no major microscopic differences were found between WT and CCR2KO, even by histological analysis, CCR2KO strain presents an impaired resolution of the inflammatory process, as demonstrated by the persistence of higher Ly6g-Gr1+ and CD68+ cells counts until late time points in CCR2KO. Accordingly, the recruitment of neutrophils to fracture sites is also increased in CCR2KO mice compared with WT mice, resulting in an altered composition of inflammatory infiltrate (10). Furthermore, it has been demonstrated that macrophages contribute to promote neutrophil clearance during early resolution phase post liver injury (33), which could explain the persistence of these cells in alveolar sockets of CCR2KO mice.

In the view of the contrasting data regarding CCR2 involvement in intramembranous and endochondral bone healing, we next performed a molecular analysis targeting multiple inflammation and healing related molecules to explore the possible impact of CCR2 lack with a highly sensitive and accurate method. From the molecular viewpoint, CCR2KO mice presented a significantly higher mRNA expression of ECM remodeling markers (Col1a2, Mmp1a, Mmp2, and Mmp9) and at 14 and 21 days, and RANKL at 21 days. On the other hand, mRNA expression of some growth factors (TGFb1) and osteoblast differentiation (Runx-2) markers was downregulated in CCR2KO mice when compared with WT mice (Figure 6). Interestingly, while the variation in healing and bone markers expression does not seems to follow a clear (upregulation or downregulation) pattern, the expression of MSC markers (CXCL12, CD106, OCT-4, NANOG, and CD146), presented a homogenous decrease in CCR2KO strain at 7 days (Figure 6). Accordingly, CCR2/CCL2 axis is essential for MSCs recruitment in a rib fracture-healing model (6).

However, despite of the molecular differences described between healing sites from CCR2KO and WT strains, it is possible to suggest that such variation was not sufficient to promote significant alteration of the healing phenotype. At this point, we must reinforce that, as previously mentioned, the bone healing is a multi-step process that involves numerous mediators and cell types playing beneficial functions along each healing step (7, 34). In this context, alterations in the migration pattern of a given cell type, suggested as macrophages in this specific case, may be compensate by other elements involved the healing process. Indeed, endothelial cells, MSCs, and other different leukocytes subsets can also release growth factors and immunological mediators involved in many steps of healing, such as angiogenesis, cell proliferation, and resolution of inflammation (35–40). A similar scenario is observed when molecules related to inflammatory cell migration (i.e., inflammatory cytokines and chemokines), where comparatively analyzed during alveolar bone healing in CCR2KO and WT strains. While the expression of some chemokines (CCL12, CCL20, and CCL25), CCR5, and the key inflammatory cytokine TNF expression was decreased in CCR2KO mice at 7 days, the expression of IL6 and CXCR1 (a receptor involved in PMN migration), where significantly higher in CCR2KO strain in the same experimental period, reinforcing that immune system compensatory mechanisms may operate in the absence of CCR2. Accordingly, macrophages are regarded as the major source of TNF during inflammation, where this cytokine plays a pivotal role regulating the pro-inflammatory response (41). However, it has been recognized that IL-6 has many pro-inflammatory functions, such as activation of the immune system, leukocyte chemoattraction (35), and also can contribute to healing processes (14, 37).

In the immunological compensation context, we also must consider that, despite a significant reduction in F4/80+ counts due the lack of CCR2, F4/80+ cells were still present in the bone healing sites in a number enough to support a proper healing outcome. In accordance, in a model of intramembranous bone healing using a model of fracture in tibia, the density of F4/80+ macrophages, as well the bone healing were not compromised by CCR2 deficiency (41). Despite the similar bone healing outcome, at this point, it is important to consider that the reduced F4/80+ cell migration may account for the impaired resolution of the inflammatory process in CCR2KO, which is accordance with the pro-resolutive role of macrophages, which include neutrophil clearance (33). However, when macrophages were aggressively depleted in the site of injury (either using the macrophage-Fas-induced apoptosis or clodronate liposome delivery mouse model), the intramembranous bone healings was drastically impaired in the same tibia fracture model (15). Despite of substantial differences between endochondral long bones and intramembranous craniofacial bones, these evidences suggest that macrophages are not only recruited *via* a CCR2-dependent mechanism.

In this way, the immunological system exhibit intrinsic features that may supply the absence of missing molecules or receptors along inflammatory/immune responses, as the redundancy developed by several cytokines and chemokine/chemokine receptor system (30, 42). Accordingly, immunofluorescence demonstrated a colocalization of CCR2 and CCR5 in macrophages in WT mice, suggesting that macrophages co-express such receptors. Indeed, the FACS analysis confirmed that 70% of F4/80+ CCR2+ cells are also CCR5+ (Figure 7) reinforcing the double-positive nature of such cells for CCR2 and CCR5 receptors; which is in line with a previous description that F4/80+ inflammatory macrophages extracted from periodontal tissues are double positive for CCR2 and CCR5 receptors (30). Interestingly, the dual CCR2/CCR5 inhibition with Cenicriviroc significantly inhibited the migration of macrophages in an acute liver injury model (43). Therefore, considering the potential co-expression of CCR2 and CCR5 in F4/80+ cells, the assumption that the dual CCR2/CCR5 inhibition could have a similar effect in bone healing process sounds plausible. In this way, the present results from CCR2KO mice draws the attention to the necessity of future studies with simultaneous inhibition of CCR2 and CCR5 along intramembranous bone healing in craniofacial bones. However, in the view of the potential involvement of other macrophage subsets (which remain to be determined by studies with specific focus in a broad phenotypic analysis of such cell in the healing sites) in intramembranous alveolar bone healing, as well of the other chemokine receptors, additional studies are required to determine the whole contribution of chemokine system to cell migration and its impact in bone healing outcome.

## Conclusion

**Figure 8:**
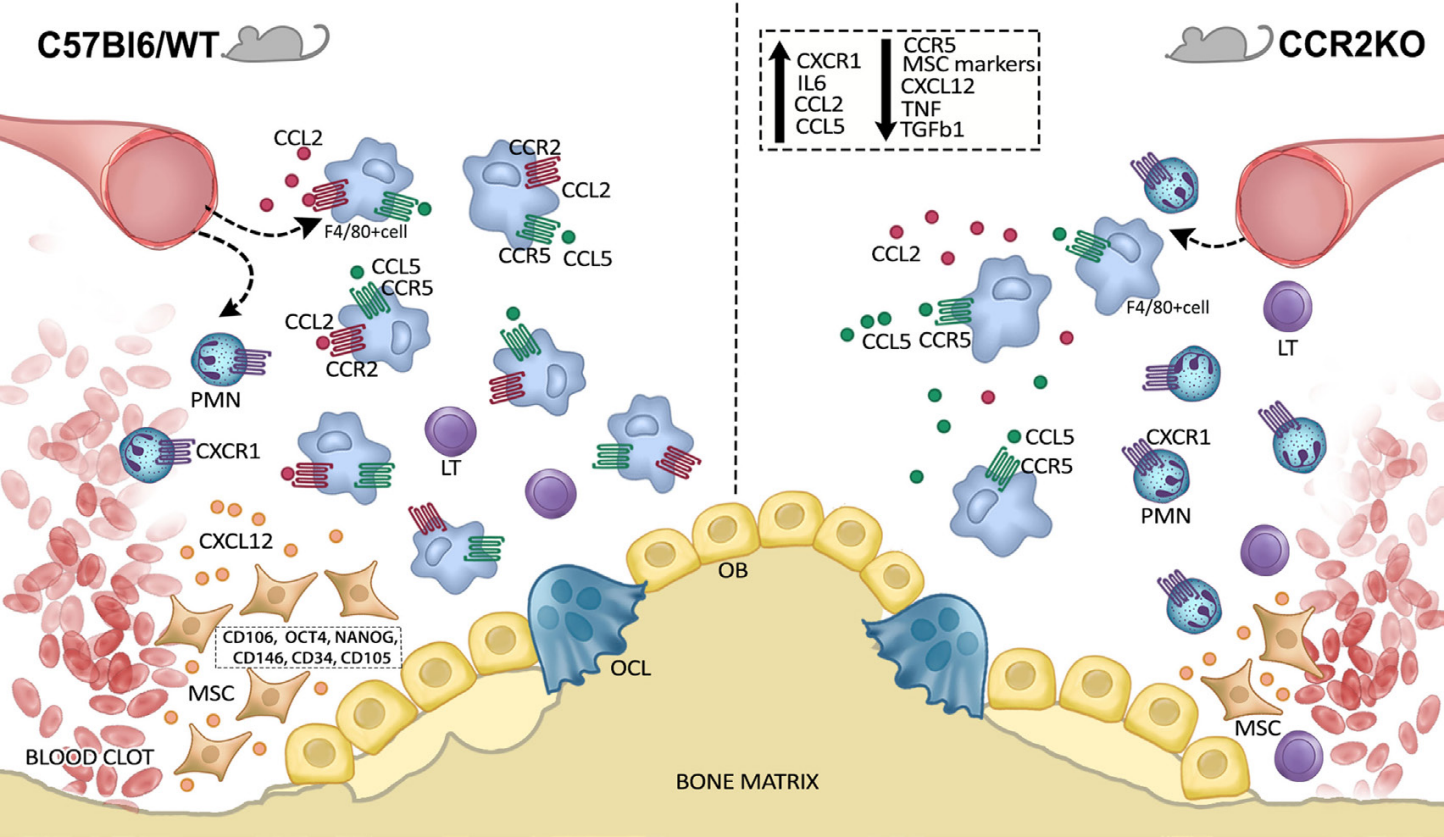
Graphical abstract of CCR2 contributions on F4/80+ cell migration along intramembranous bone healing in maxilla. CCR2 contributes to monocytes/macrophages into alveolar bone injury post tooth extraction, and consequently result in downregulation of mesenchymal stem cell (MSC) markers and growth factors at the healing sites. On the other hand, the migration of the F4/80+ cells in CCR2KO mice it is enough to support the proper healing, in a scenario that can involve compensatory immunological mechanisms.

Our results indicate that CCR2 plays an active role on F4/80+ cells migration after alveolar bone injury, and consequently result in downregulation of MSC markers and growth factors at the healing sites (Figure 8). However, since CCR2 absence does not significantly impact the outcome of intramembranous bone healing at the endpoint, it is reasonable to suggest that, although reduced, the migration of the F4/80+ cells in CCR2KO mice it is enough to support the proper healing, in a scenario that can involve compensatory immunological mechanisms.

## Ethics Statement

This study was carried out in strict accordance with the recommendations in the Guide for the Care and Use of Laboratory Animals of the National Institutes of Health. The experimental protocol was approved by the local Institutional Committee for Animal Care and Use (Committee on Animal Research and Ethics CEEPA-FOB/USP, process #005/2012).

## Author Contributions

CB: contributed to acquisition, analysis, and interpretation; drafted manuscript; critically revised manuscript; gave final approval; agreed to be accountable for all aspects of work. AV, FC, and AF: contributed to acquisition, analysis, and interpretation; critically revised manuscript; gave final approval; agreed to be accountable for all aspects of work. PC: contributed to acquisition and analysis; critically revised manuscript; gave final approval; agreed to be accountable for all aspects of work. RS: contributed to conception, contributed to acquisition and interpretation; critically revised manuscript; gave final approval; agreed to be accountable for all aspects of work. AT: contributed to conception and design, contributed to analysis and interpretation; critically revised manuscript; gave final approval; agreed to be accountable for all aspects of work. GG: contributed to conception and design; contributed to acquisition, analysis, and interpretation; drafted manuscript; critically revised manuscript; gave final approval; agreed to be accountable for all aspects of work.

## Conflict of Interest Statement

The authors declare that the research was conducted in the absence of any commercial or financial relationships that could be construed as a potential conflict of interest.
